# Effects of ganglioside GM1 and erythropoietin on spinal cord injury in mice: Functional and immunohistochemical assessments

**DOI:** 10.1016/j.clinsp.2022.100006

**Published:** 2022-02-19

**Authors:** Alessandro Gonzalez Torelli, Alexandre Fogaça Cristante, Tarcísio Eloy Pessoa de Barros-Filho, Gustavo Bispo dos Santos, Beatriz Cintra Morena, Felipe Fernandes Correia, Vera Paschon

**Affiliations:** a Divisão de Cirurgia de Coluna Vertebral, Instituto de Ortopedia e Traumatologia (IOT), Hospital das Clínicas da Faculdade de Medicina da Universidade de São Paulo (HCFMUSP), São Paulo, SP, Brazil; b Laboratório de Investigação Médica (LIM 41), Faculdade de Medicina da Universidade de São Paulo (FMUSP), São Paulo, SP, Brazil; c Laboratório de Neurogenética, Universidade Federal do ABC, Santo Andre, SP, Brazil

**Keywords:** Erythropoietin, GM1 ganglioside, Spinal cord compression, Spinal cord, Mice

## Abstract

**Objectives:**

To evaluate the functional and immunohistochemical effects of ganglioside GM1 and erythropoietin following experimental spinal cord injury.

**Methods:**

Thirty-two male BALB/c mice were subjected to experimental spinal cord injury using the NYU Impactor device and were randomly divided into the following groups: GM1 group, receiving standard ganglioside GM1 (30 mg/kg); erythropoietin group, receiving erythropoietin (1000 IU/kg); combination group, receiving both drugs; and control group, receiving saline (0.9%). Animals were evaluated according to the Basso Mouse Scale (BMS) and Hindlimb Mouse Function Score (MFS). After euthanasia, the immunohistochemistry of the medullary tissue of mice was analyzed. All animals received intraperitoneal treatment.

**Results:**

The GM1 group had higher BMS and MFS scores at the end of the experiment when compared to all other groups. The combination group had higher BMS and MFS scores than the erythropoietin and control groups. The erythropoietin group had higher BMS and MFS scores than the control group. Immunohistochemical tissue analysis showed a significant difference among groups. There was a significant increase in myelinated axons and in the myelinated axon length in the erythropoietin group when compared to the other intervention groups (p < 0.01).

**Conclusions:**

Erythropoietin and GM1 have therapeutic effects on axonal regeneration in mice subjected to experimental spinal cord injury, and administration of GM1 alone had the highest scores on the BMS and MFS scales.

## Highlights

• GM1 and erythropoietin bring neurological improvement in mice with blunt spinal cord injury.

## Introduction

Functional loss due to spinal cord trauma is the end result of a continuous multifactorial process divided into two main stages. In the first stage, initiated at the injury site, the transfer of mechanical energy to the tissue causes cell death, promoting necrosis.^1^ In the second stage, following the immediate injury, there is a secondary injury that affects the injury site and the adjacent tissue, leading to apoptosis.^2^^,^^3^

Secondary injury is associated with metabolic changes that begin immediately after the initial injury. These changes include activation of the arachidonic acid cascade, inflammatory response, production of reactive oxygen species, and increased extracellular concentration of glutamate, leading to edema and reduced blood flow to the spinal cord.^4^, ^5^, ^6^

The benefits of GM1 in the treatment of secondary neurological injuries caused by stroke, diabetic peripheral neuropathy, and Parkinson's disease has been shown in several studies.^7^^,^^8^ The mechanisms of action of GM1 are believed to reduce neuronal edema by increasing the activity of ion pumps, favoring cell homeostasis^9^ and, mainly, promoting the increase of endogenous protective factors. This action reduces the intensity of secondary cell damage after the initial trauma, intensifies the adaptive mechanisms of neural plasticity, and promotes new synapses among neurons, possibly resulting in functional improvement.^10^

On the other hand, erythropoietin, a glycoprotein mainly produced in the kidney of adults, can promote cellular protection in several tissues, including nervous tissue. According to the current literature, the main effects attributed to this glycoprotein are the promotion of apoptosis blockade, modulation of events in the inflammatory cascade, protection, and optimization of microvascular repair, and neuronal regeneration.^11^^,^^12^

Erythropoietin has a neuroprotective action and has been studied in the treatment of spinal cord trauma.^13^, ^14^, ^15^, ^16^, ^17^ Erythropoietin itself and its receptors are present in the human brain, especially in developing tissues. However, their action mechanisms are not well known yet.^18^

There has been a long search for a way to accelerate or improve the natural neuronal regeneration process. Recent biomolecular studies have set the path in this search by identifying the role of GM1 and erythropoietin in experimental models of nerve regeneration, but the number of studies with proven potential for clinical application is still limited.

In this sense, the present study was motivated by the possibility of applying GM1 combined with erythropoietin in the treatment of Spinal Cord Injury (SCI) in mice, as these substances may represent an advance in the quality of nerve regeneration.

The quality of life of patients with SCI can be significantly improved with minimal anatomical recovery because the spinal cord does not necessarily need to be completely reconstructed to positively impact the quality of life of these patients.

This study aims to evaluate the functional and immunohistological effect of treatment with GM1 and erythropoietin, alone and in combination, as agents that promote neural protection and regeneration in spinal cord contusion injury in BALB/c mice.

## Methods

### Ethics

The study protocol was evaluated and approved by the Research Ethics Committee of the institution (Ethics Committee on the Use of Animals – CEUA, Medical School of Universidade de São Paulo – USP; authorization number: 072/16).

All institutional guidelines ruling research involving animals were followed. These guidelines are in accordance with those of international scope regulating pain control in research involving experimental animals.^19^

During the study, all animals were kept in climatized cages and under proper hygiene, feeding, and hydration conditions. Cages were stored in the laboratory. Each cage, whose dimensions were 30 × 19.8 × 13.3 cm, contained up to three mice from the same litter. Animals were often handled by laboratory caregivers with the aim of getting them used to the laboratory technical staff and conditioning them to their regular movement, allowing for greater ease in subsequent motor capacity assessments following experimental injury.

### Study design, sample size, and experimental animals

In this controlled study, BALB/c mice were divided into four groups. The sample size was based on previously published studies, using eight to 10 animals per group.^20^^,^^21^

Thirty-two BALB/c mice were included in this study. Inclusion criteria were male animals weighing 70 to 100 g and aged between 10 and 12 weeks. All animals should have a normal coat, normal clinical status, and normal mobility conditions. The purpose of this assessment was to ensure that all animals were healthy and had a normal movement capability at baseline. All animals were weighed at the beginning and at the end of the study.

At first contact, all mice were inspected by the researcher and by the veterinarian responsible for the laboratory to assess their general condition.

In this sense, exclusion criteria were:

a) Death following experimental spinal cord trauma;
b) Malformations or anatomical anomalies macroscopically observed in the injured spinal cord area;
c) Autophagy or mutilation among animals during the observation period;
d) Normal movement in the first post-injury assessment – nine points on the Basso Mouse Scale (BMS), which represent a failure in the experimental SCI; and
e) Surgical site infection.

Mice were distributed by simple drawing into four groups with eight animals each, all of them being submitted to experimental SCI as described below:

a) Control group – mice submitted to SCI and intraperitoneal administration of saline (0.9%);
b) GM1 group – mice submitted to SCI and intraperitoneal administration of ganglioside GM1 (30 mg/kg);
c) Erythropoietin group – mice submitted to SCI and intraperitoneal administration of erythropoietin (1000 IU/kg); and
d) Combination group – mice submitted to SCI and combined intraperitoneal administration of GM1 (30 mg/kg) and erythropoietin (1000 IU/kg).

### Procedures

For anesthesia, the association of 90 mg/kg of ketamine and 5 mg/kg of xylazine was used. The absence of corneal reflexes and absence of reaction to tail and hind limb pinch compression confirmed the maintenance of the anesthetic plane. The protocol provides for the administration of one-third of the initial dose as an anesthetic booster.^22^^,^^23^

Following the anesthetic plan confirmation, each animal underwent laminectomy at the T9 level, with subsequent exposure of the spine. The entire surgical procedure was performed with the aid of a surgical microscope, thus minimizing the risk of inadvertent SCI.

To perform the spinal cord injuries, the international protocol Multicenter Animal Spinal Cord Injury Study (MASCIS)^24^ was followed. Injuries were obtained using the NYU Impactor system.^22^^,^^25^

A moderate spinal contusion injury was induced at T9 level as previously described,^26^ using an NYU Impactor device with an 8g and 12.5 mm impact rod to compress the spinal cord. This procedure caused an SCI with consequent loss of locomotor function.

After the surgical procedure, animals were transferred to their respective cages, receiving food and water ad libitum. Wood shavings were changed, and proper cage cleaning was performed on a regular basis.

Given the risk of SCI-associated urinary retention, bladder manipulation was performed daily for five days through massage for bladder emptying every eight hours. Throughout the experiment, animals were monitored and examined in order to identify complications such as self-mutilation and urinary or surgical site infection.

For analgesia and antibiotic prophylaxis, all animals received buprenorphine at a dose of 0.01 to 0.05 mg/kg subcutaneously. Amoxicillin was administered at a dose of 15 mg/kg intraperitoneally every 12 hours. All medications were administered in all groups, including the control group.

All mice were kept in the same vivarium and under the same controlled environmental conditions for 42 days. For the euthanasia procedure, animals became unconscious under anesthesia and had a painless death through the infusion of 5 mL of potassium chloride solution (19.1%) intravenously.

The spinal cords were extracted and placed in properly identified Falcon tubes containing 30 mL of 10% sucrose solution at room temperature, being kept in this solution for up to 24 hours. Then, they were sent to the Neurogenetics Laboratory for immunohistochemistry preparation.

### Functional assessment

Motor function was assessed using the BMS and Hindlimb Mouse Function Score (MFS) rating scales.^25^, ^26^, ^27^ The BMS scale ranges from complete paraplegia (score 0) to normal neurological function (score 9), while the MFS scale ranges from complete paraplegia to normal neurological function (score 13). The observation of animals was carried out by two trained researchers blinded as to which group each animal belonged to and also as to the evaluation of their peers. After the intervention, serial assessments were carried out for 5 minutes at 2, 14, 28, and 42 days after the experimental SCI. Outcomes were classified by the responsible researchers according to the BMS and MFS scales. In cases of disagreement, the lowest score was recorded for analysis.

### Statistical analysis

The aim of this study is to verify if there are differences in the parameters of interest set among the groups at each assessment time point, as well as if there is any difference in the immunohistochemistry result among the experimental groups.

The scales adapted before the injury were extracted per group by means of summarized measures (mean, standard deviation, median, minimum, and maximum) and compared among groups using analysis of variance (ANOVA). Immunohistochemical measures were assessed per group using abstract measures and compared among groups using Generalized Linear Models with Poisson distribution (GLM), link function for the number of axons, with normal distribution, and logarithmic link function for the sum of axon lengths. Analyses were followed by Bonferroni multiple comparisons to identify which groups were different.

Function parameters were described per group throughout the follow-up period and compared among groups and assessment time points and using Generalized Estimating Equations (GEE) with normal distribution and identity link function, assuming a first-order autoregressive correlation matrix among time points. Results were subjected to Bonferroni multiple comparisons to identify groups and time points showing relevant differences.

Analyses were performed using IBM-SPSS software for Windows version 20.0 and tabulated in the software Microsoft Excel 2003. The significance level of performed tests was 5%.

## Results

*Functional assessment using the BMS and MFS scales*

During follow-up and assessment, none of the mice were excluded from the study.

All four groups showed increased scores on both assessments scales over the six-week period of the study. There was no statistically significant score difference between the right and left limbs of animals (p > 0.05). Data are summarized in Table 1.

**Table 1:** Description of functional scales before injury per group and results of comparative tests.

**Variable**	**Control**	**GM1**	**Erythropoietin**	**GM1 + Erythropoietin**	**p**
	**(n = 8)**	**(n = 8)**	**(n = 8)**	**(n = 8)**	
**BMS**					>0.999
Mean ± DP	9±0	9±0	9±0	9±0	
Median (min–max)	9 (9–9)	9 (9–9)	9 (9–9)	9 (9–9)	
**MFS**					0.451
Mean ± DP	11.4±1.2	10.5±1.1	11±1.1	11±0.9	
Median (min–max)	11 (10–13)	11 (9–12)	11 (10–13)	11 (10–13)	

ANOVA

**Fig. 1:**
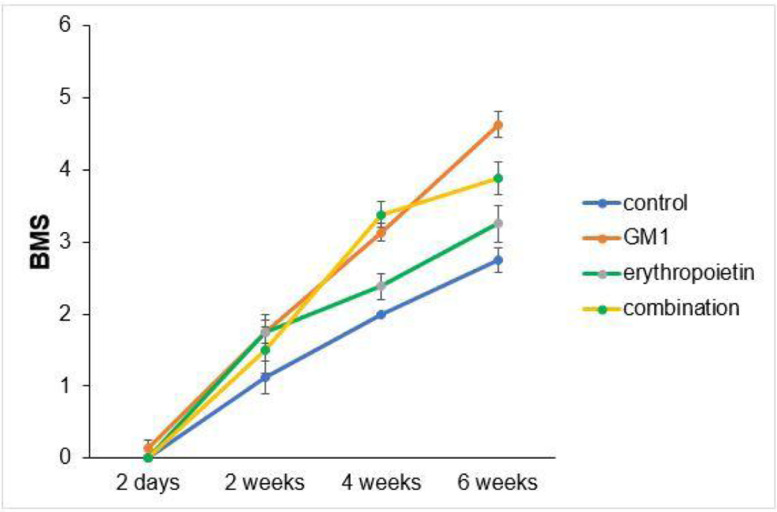
BMS scale mean profiles per group and respective standard errors.

**Fig. 2:**
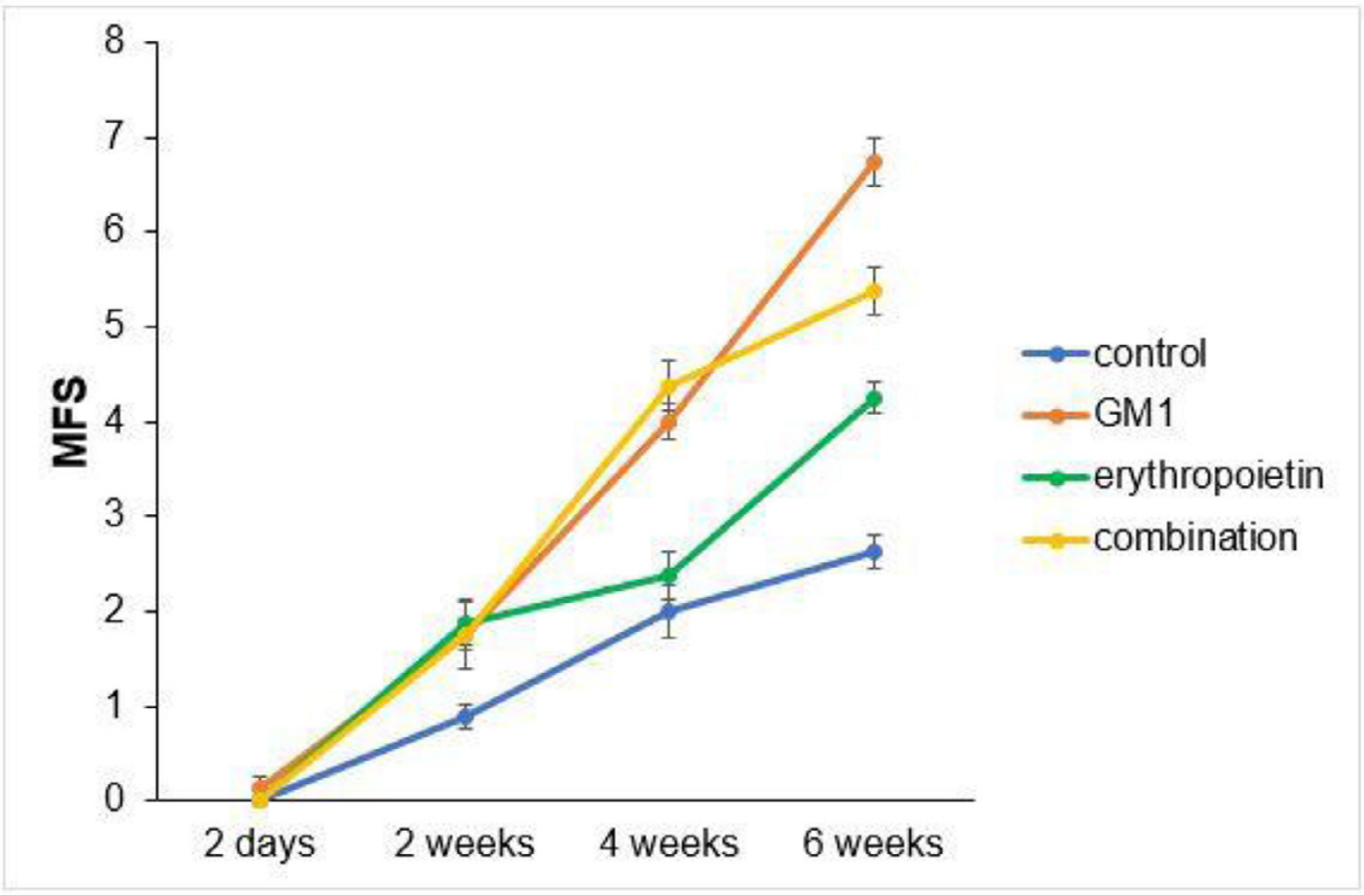
MFS scale mean profiles per group and respective standard errors.

Scores obtained in serial assessments over the six weeks of the experiment suggest that animals that received GM1 showed increased scale values more quickly than animals that received saline (control) or erythropoietin alone, as shown in Fig. 1, Fig. 2.

Both on the BMS scale and on the MFS scale, the average behavior of animals during follow-up was statistically different among groups (p-interaction < 0.001), as detailed in Table 2, corroborating the findings suggested in Figs. 1 and 2.

**Table 2:** Description of functional scales per group and assessment time point after injury induction and results of comparative tests.

**Variable/Group**	**Evaluation schedule**				**p-group**	**p-moment**	**p-interaction**
	**2-days**	**2-weeks**	**4-weeks**	**6-weeks**			
**BMS**					< 0.001	< 0.001	< 0.001
**Control**							
Mean ± DP	0 ± 0	1.1 ± 0.6	2 ± 0	2.8 ± 0.5			
Median (min–max)	0 (0–0)	1 (0–2)	2 (2–2)	3 (2–3)			
**GM1**							
Mean ± DP	0.1 ± 0.4	1.8 ± 0.5	3.1 ± 0.4	4.6 ± 0.5			
Median (min–max)	0 (0–1)	2 (1–2)	3 (3–4)	5 (4–5)			
**Erythropoietin**							
Mean ± DP	0 ± 0	1.8 ± 0.7	2.4 ± 0.5	3.3 ± 0.7			
Median (min–max)	0 (0–0)	2 (1–3)	2 (2–3)	3 (2–4)			
**GM1+Erythropoietin**							
Mean ± DP	0 ± 0	1.5 ± 0.9	3.4 ± 0.5	3.9 ± 0.6			
Median (min–max)	0 (0–0)	1,5 (0–3)	3 (3–4)	4 (3–5)			
**MFS**					< 0.001	< 0.001	< 0.001
**Control**							
Mean ± DP	0 ± 0	0.9 ± 0.4	2 ± 0.8	2.6 ± 0.5			
Median (min–max)	0 (0–0)	1 (0–1)	2 (1–3)	3 (2–3)			
**GM1**							
Mean ± DP	0.1 ± 0.4	1.8 ± 0.5	4 ± 0.5	6.8 ± 0.7			
Median (min–max)	0 (0–1)	2 (1–2)	4 (3–5)	7 (5–7)			
**Erythropoietin**							
Mean ± DP	0 ± 0	1.9 ± 0.6	2.4 ± 0.7	4.3 ± 0.5			
Median (min–max)	0 (0–0)	2 (1–3)	2 (2–4)	4 (4–5)			
**GM1+Erythropoietin**							
Mean ± DP	0 ± 0	1.8 ± 1	4.4 ± 0.7	5.4 ± 0.7			
Median (min–max)	0 (0–0)	2 (0–3)	4.5 (3–5)	5.5 (4–6)			

EEG with normal distribution and identity binding function with AR (1) correlation matrix between moments.

Mean differences among groups, on both scales, only started to occur after four weeks of assessment. In the groups that received GM1 alone or combined with erythropoietin, these differences were greater than those observed in the control group at four weeks and greater than those observed in the control group and in the erythropoietin group at six weeks (p < 0.05), with small differences between the scales. During follow-up, the only group that showed a mean increase in scales at all assessment time points was GM1 (p < 0.05); the other groups also showed such an increase throughout the assessment time points, but the increase was not always statistically significant, mainly between two consecutive assessments. For example, in the erythropoietin group, from the second to the fourth week, there was no statistically significant mean increase in any of the scales (p > 0.999). Details of these findings are presented in Table 3 and in Table 4.

**Table 3:** Result of multiple BMS scale comparisons per group and assessment time point.

**BMS**							
**Moment/ Group**	**Comparisons**	**Mean difference**	**Standard deviation**	**gl**	**p**	**95% IC**	
						**Inferior**	**Superior**
2-days	Control vs. GM1	-0.13	0.26	1	>0.999	-1.02	0.77
	Control vs. Erythropoietin	0.00	0.26	1	>0.999	-0.90	0.90
	Control vs. GM1+Erythropoietin	0.00	0.26	1	>0.999	-0.90	0.90
	GM1 vs. Erythropoietin	0.13	0.26	1	>0.999	-0.77	1.02
	GM1 vs. GM1+Erythropoietin	0.13	0.26	1	>0.999	-0.77	1.02
	Erythropoietin vs. GM1+Erythropoietin	0.00	0.26	1	>0.999	-0.90	0.90
2-weeks	Control vs. Gm1	-0.63	0.26	1	>0.999	-1.52	0.27
	Control vs. Erythropoietin	-0.63	0.26	1	>0.999	-1.52	0.27
	Control vs. GM1+Erythropoietin	-0.38	0.26	1	>0.999	-1.27	0.52
	GM1 vs. Erythropoietin	0.00	0.26	1	>0.999	-0.90	0.90
	GM1 vs. GM1+Erythropoietin	0.25	0.26	1	>0.999	-0.65	1.15
	Erythropoietin vs. GM1+Erythropoietin	0.25	0.26	1	>0.999	-0.65	1.15
4-weeks	Control vs. Gm1	-1.13	0.26	1	0.001	-2.02	-0.23
	Control vs. Erythropoietin	-0.38	0.26	1	>0.999	-1.27	0.52
	Control vs. GM1+Erythropoietin	-1.38	0.26	1	<0.001	-2.27	-0.48
	GM1 vs. Erythropoietin	0.75	0.26	1	0.392	-0.15	1.65
	GM1 vs. GM1+Erythropoietin	-0.25	0.26	1	>0.999	-1.15	0.65
	Erythropoietin vs. GM1+Erythropoietin	-1.00	0.26	1	0.011	-1.90	-0.10
6-weeks	Control vs. Gm1	-1.88	0.26	1	<0.001	-2.77	-0.98
	Control vs. Erythropoietin	-0.50	0.26	1	>0.999	-1.40	0.40
	Control vs. GM1+Erythropoietin	-1.13	0.26	1	0.001	-2.02	-0.23
	GM1 vs. Erythropoietin	1.38	0.26	1	<0.001	0.48	2.27
	GM1 vs. GM1+Erythropoietin	0.75	0.26	1	0.392	-0.15	1.65
	Erythropoietin vs. GM1+Erythropoietin	-0.63	0.26	1	>0.999	-1.52	0.27
Control	2-days vs. 2-weeks	-1.13	0.24	1	<0.001	-1.96	-0.29
	2-days vs. 4-weeks	-2.00	0.25	1	<0.001	-2.89	-1.11
	2-days vs. 6-weeks	-2.75	0.26	1	<0.001	-3.65	-1.85
	2-weeks vs. 4-weeks	-0.88	0.24	1	0.028	-1.71	-0.04
	2-weeks vs. 6-weeks	-1.63	0.25	1	<0.001	-2.52	-0.73
	4-weeks vs. 6-weeks	-0.75	0.24	1	0.191	-1.59	0.09
GM1	2-days vs. 2-weeks	-1.63	0.24	1	<0.001	-2.46	-0.79
	2-days vs. 4-weeks	-3.00	0.25	1	<0.001	-3.89	-2.11
	2-days vs. 6-weeks	-4.50	0.26	1	<0.001	-5.40	-3.60
	2-weeks vs. 4-weeks	-1.38	0.24	1	<0.001	-2.21	-0.54
	2-weeks vs. 6-weeks	-2.88	0.25	1	<0.001	-3.77	-1.98
	4-weeks vs. 6-weeks	-1.50	0.24	1	<0.001	-2.34	-0.66
Erythropoietin	2-days vs. 2-weeks	-1.75	0.24	1	<0.001	-2.59	-0.91
	2-days vs. 4-weeks	-2.38	0.25	1	<0.001	-3.27	-1.48
	2-days vs. 6-weeks	-3.25	0.26	1	<0.001	-4.15	-2.35
	2-weeks vs. 4-weeks	-0.62	0.24	1	>0.999	-1.46	0.21
	2-weeks vs. 6-weeks	-1.50	0.25	1	<0.001	-2.39	-0.61
	4-weeks vs. 6-weeks	-0.88	0.24	1	0.028	-1.71	-0.04
GM1 + Erythropoietin	2-days vs. 2-weeks	-1.50	0.24	1	<0.001	-2.34	-0.66
	2-days vs. 4-weeks	-3.38	0.25	1	<0.001	-4.27	-2.48
	2-days vs. 6-weeks	-3.88	0.26	1	<0.001	-4.77	-2.98
	2-weeks vs. 4-weeks	-1.88	0.24	1	<0.001	-2.71	-1.04
	2-weeks vs. 6-weeks	-2.38	0.25	1	<0.001	-3.27	-1.48
	4-weeks vs. 6-weeks	-0.50	0.24	1	>0.999	-1.34	0.34

Bonferroni multiple comparisons.

**Table 4:** Result of multiple MFS scale comparisons per group and assessment time point.

**MFS**							
**Moment/ Group**	**Comparisons**	**Mean difference**	**Standard deviation**	**gl**	**p**	**95% IC**	
						**Inferior**	**Superior**
2-days	Control vs. GM1	-0.13	0.29	1	>0.999	-1.15	0.90
	Control vs. Erythropoietin	0.00	0.29	1	>0.999	-1.03	1.03
	Control vs. GM1+Erythropoietin	0.00	0.29	1	>0.999	-1.03	1.03
	GM1 vs. Erythropoietin	0.13	0.29	1	>0.999	-0.90	1.15
	GM1 vs. GM1+Erythropoietin	0.13	0.29	1	>0.999	-0.90	1.15
	Erythropoietin vs. GM1+Erythropoietin	0.00	0.29	1	>0.999	-1.03	1.03
2-weeks	Control vs. Gm1	-0.88	0.29	1	0.325	-1.90	0.15
	Control vs. Erythropoietin	-1.00	0.29	1	0.073	-2.03	0.03
	Control vs. GM1+Erythropoietin	-0.88	0.29	1	0.325	-1.90	0.15
	GM1 vs. Erythropoietin	-0.13	0.29	1	>0.999	-1.15	0.90
	GM1 vs. GM1+Erythropoietin	0.00	0.29	1	>0.999	-1.03	1.03
	Erythropoietin vs. GM1+Erythropoietin	0.13	0.29	1	>0.999	-0.90	1.15
4-weeks	Control vs. Gm1	-2.00	0.29	1	<0.001	-3.03	-0.97
	Control vs. Erythropoietin	-0.38	0.29	1	>0.999	-1.40	0.65
	Control vs. GM1+Erythropoietin	-2.38	0.29	1	<0.001	-3.40	-1.35
	GM1 vs. Erythropoietin	1.63	0.29	1	<0.001	0.60	2.65
	GM1 vs. GM1+Erythropoietin	-0.38	0.29	1	>0.999	-1.40	0.65
	Erythropoietin vs. GM1+Erythropoietin	-2.00	0.29	1	<0.001	-3.03	-0.97
6-weeks	Control vs. Gm1	-4.13	0.29	1	<0.001	-5.15	-3.10
	Control vs. Erythropoietin	-1.63	0.29	1	<0.001	-2.65	-0.60
	Control vs. GM1+Erythropoietin	-2.75	0.29	1	<0.001	-3.78	-1.72
	GM1 vs. Erythropoietin	2.50	0.29	1	<0.001	1.47	3.53
	GM1 vs. GM1+Erythropoietin	1.38	0.29	1	<0.001	0.35	2.40
	Erythropoietin vs. GM1+Erythropoietin	-1.13	0.29	1	0.014	-2.15	-0.10
Control	2-days vs. 2-weeks	-0.88	0.28	1	0.244	-1.88	0.13
	2-days vs. 4-weeks	-2.00	0.29	1	<0.001	-3.03	-0.97
	2-days vs. 6-weeks	-2.63	0.29	1	<0.001	-3.65	-1.60
	2-weeks vs. 4-weeks	-1.13	0.28	1	0.009	-2.13	-0.12
	2-weeks vs. 6-weeks	-1.75	0.29	1	<0.001	-2.78	-0.72
	4-weeks vs. 6-weeks	-0.63	0.28	1	>0.999	-1.63	0.38
GM1	2-days vs. 2-weeks	-1.62	0.28	1	<0.001	-2.63	-0.62
	2-days vs. 4-weeks	-3.88	0.29	1	<0.001	-4.90	-2.85
	2-days vs. 6-weeks	-6.63	0.29	1	<0.001	-7.65	-5.60
	2-weeks vs. 4-weeks	-2.25	0.28	1	<0.001	-3.25	-1.25
	2-weeks vs. 6-weeks	-5.00	0.29	1	<0.001	-6.03	-3.97
	4-weeks vs. 6-weeks	-2.75	0.28	1	<0.001	-3.75	-1.75
Erythropoietin	2-days vs. 2-weeks	-1.88	0.28	1	<0.001	-2.88	-0.87
	2-days vs. 4-weeks	-2.38	0.29	1	<0.001	-3.40	-1.35
	2-days vs. 6-weeks	-4.25	0.29	1	<0.001	-5.28	-3.22
	2-weeks vs. 4-weeks	-0.50	0.28	1	>0.999	-1.50	0.50
	2-weeks vs. 6-weeks	-2.38	0.29	1	<0.001	-3.40	-1.35
	4-weeks vs. 6-weeks	-1.88	0.28	1	<0.001	-2.88	-0.87
GM1 + Erythropoietin	2-days vs. 2-weeks	-1.75	0.28	1	<0.001	-2.75	-0.75
	2-days vs. 4-weeks	-4.38	0.29	1	<0.001	-5.40	-3.35
	2-days vs. 6-weeks	-5.38	0.29	1	<0.001	-6.40	-4.35
	2-weeks vs. 4-weeks	-2.63	0.28	1	<0.001	-3.63	-1.62
	2-weeks vs. 6-weeks	-3.63	0.29	1	<0.001	-4.65	-2.60
	4-weeks vs. 6-weeks	-1.00	0.28	1	0.051	-2.00	0.00

Bonferroni multiple comparisons

### Immunohistochemical analysis

Treatment with erythropoietin increases axonal remyelination six weeks after SCI.

**Fig. 3:**
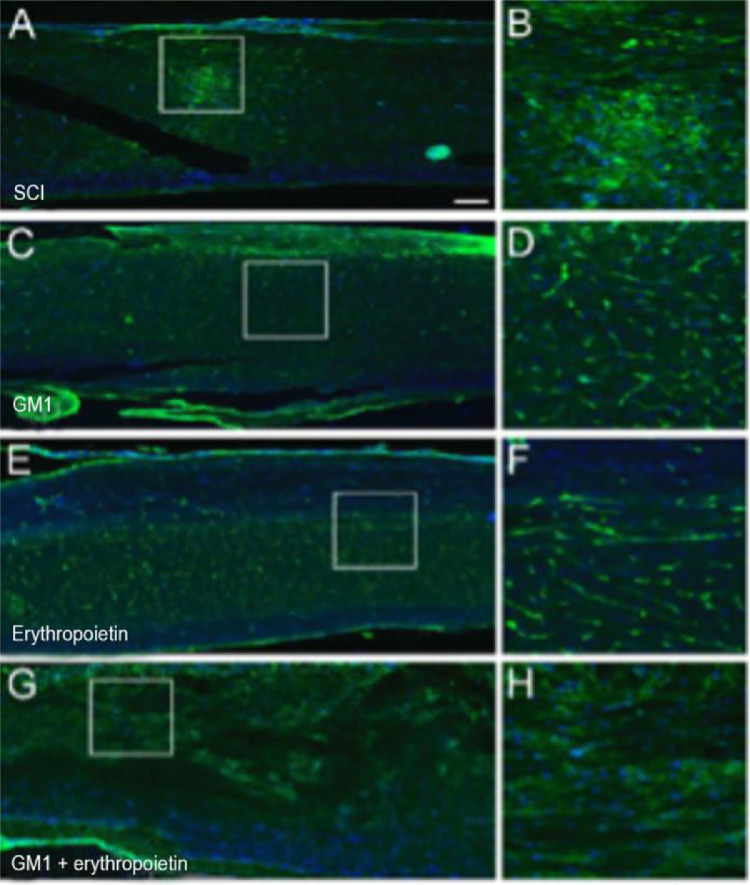
(A) Myelin Basic Protein (MBP, green) immunofluorescence counterstained with 4′, 6-Diamidino-2-Phenylindole (DAPI, blue) in longitudinal spinal cord sections of mice submitted to spinal cord injury (control group). (B) Microphotograph of the spinal cord of an animal in control group labeled with MBP. (C) Digital zoom of the area selected in A. (D) Microphotograph of the spinal cord of an animal with SCI treated with GM1. (E) Digital zoom of the area selected in C. (F) Microphotograph of the spinal cord of an animal with SCI treated with erythropoietin. (G) Digital zoom of the area selected in E. (H) Microphotograph of the spinal cord of an animal with SCI treated with a GM1 + erythropoietin combination.

**Fig. 4:**
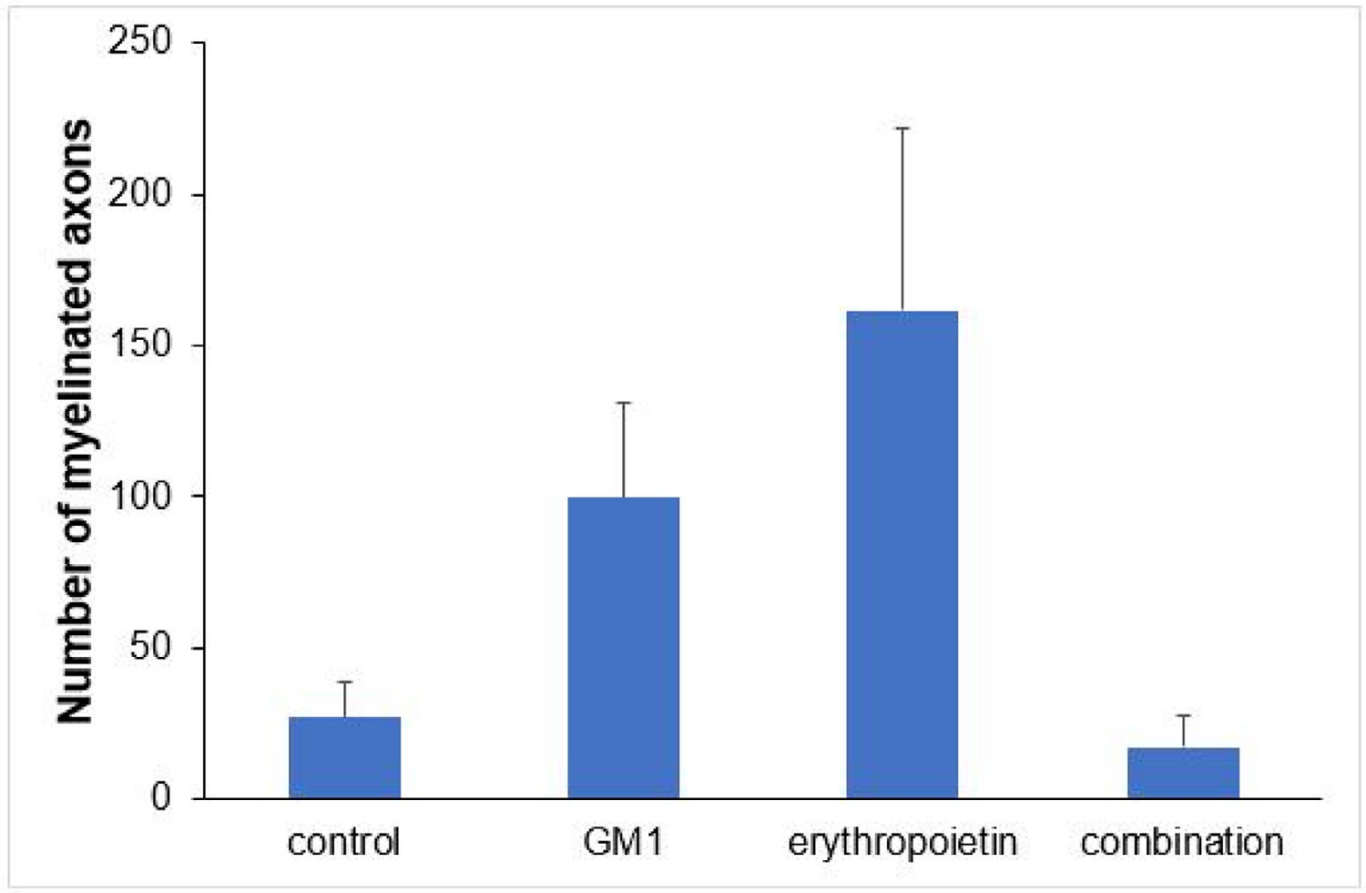
Mean values and respective standard errors of the number of axons per group.

**Fig. 5:**
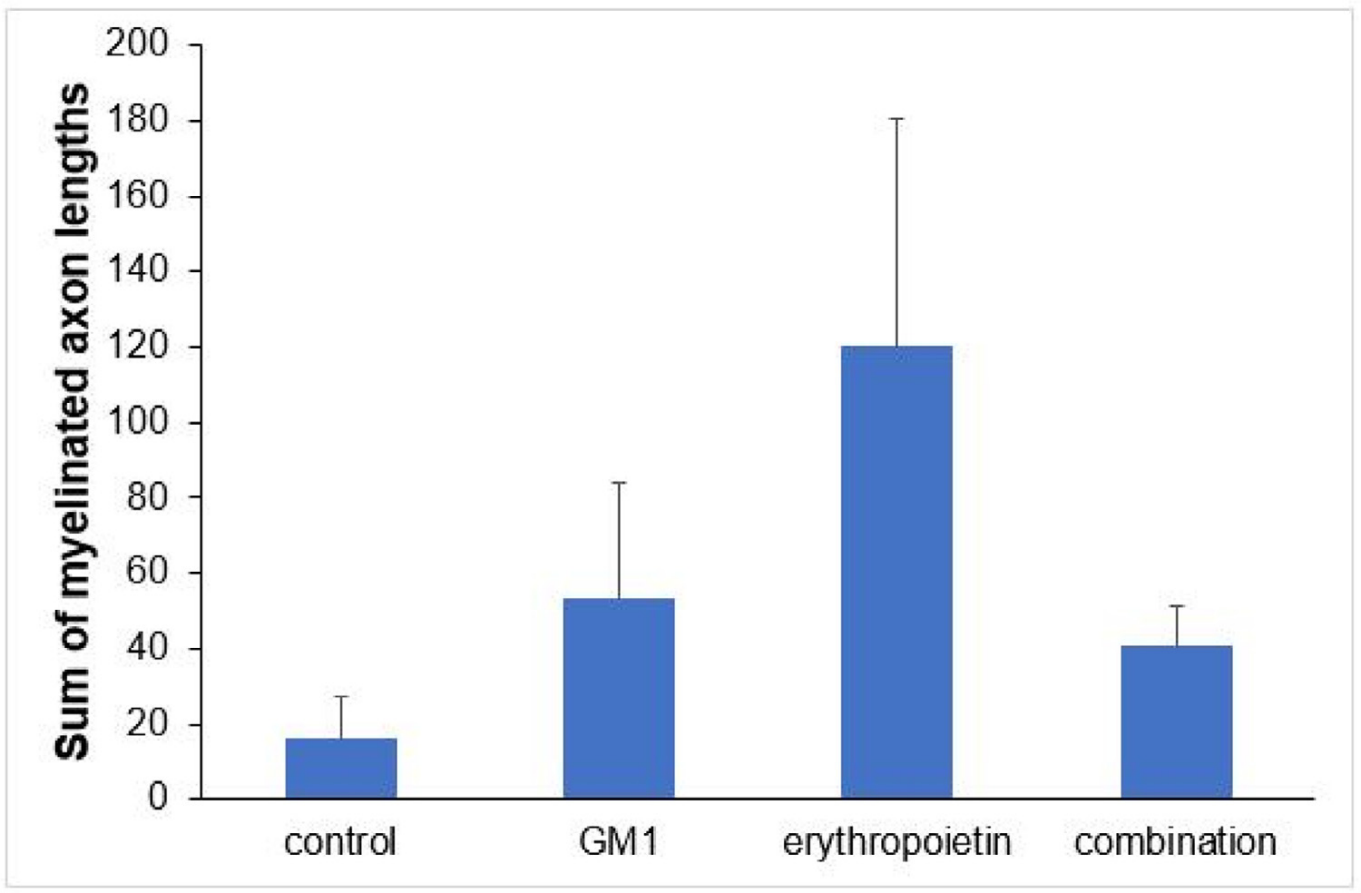
Mean values and respective standard errors of the sum of axon lengths per group.

Using a specific antibody to Myelin Basic Protein (MBP), the authors assessed axonal remyelination six weeks after SCI. As shown in Fig. 3 (A–H), longitudinal spinal cord sections of mice submitted to SCI and saline solution (control group), treated with GM1, treated with erythropoietin, and treated with a combination of GM1 + Erythropoietin were labeled with MBP and counterstained with 4′, 6-diamidino-2-phenylindole (DAPI). Using the NeuroJ plugin developed for the ImageJ image analysis program, the number of myelinated axons was estimated to be approximately 27.40 ± 11.42 for the control group, 99.8 ± 31.12 for the GM1 group, 161.60 ± 60.30 for the erythropoietin group, and 6.40 ± 10.65 for the combination group. The erythropoietin group was significantly different from the control and combination groups, as shown in Fig. 4 (n = 5, p < 0.05). The sum of the length of myelinated axons was quantified using the same software, being approximately 15.97 ± 5.62 inches for the control group, 53.23 ± 6.54 inches for the GM1 group, 120.14 ± 34.38 inches for the erythropoietin group, and 39.31 ± 31.02 inches for the combination group. Again, the erythropoietin group was significantly different from the control groups, as shown in Fig. 5 (n = 5, p < 0.05).

Fig. 4 suggests that are fewer myelinated axons in the control and combination groups and more axons in the erythropoietin group.

Fig. 5 shows the sum of myelinated axon lengths was lower in the control group and higher in the erythropoietin group.

Table 5 shows that, on average, there were statistically significant differences both in the number of myelinated axons and the sum of the lengths of myelinated axons among groups (p<0.001 and p = 0.024, respectively).

**Table 5:** Description of immunohistochemical parameters per group and results of comparative tests.

**Variable**	**Control**	**GM1**	**Erythropoietin**	**GM1 + Erythropoietin**	**p**
	**(n = 8)**	**(n = 8)**	**(n = 8)**	**(n = 8)**	
Number of myelinated axons					<0.001
Mean ± DP	27.4±25.5	99.8±69.6	161.6±134.8	17.2±23.8	
Median (min–max)	21 (2–59)	110 (31–205)	123 (19–330)	9 (1–59)	
Sum of myelinated axon lengths					0.024^a^
Mean ± DP	16±12.6	53.2±14.6	120.1±76.9	40.6±60.2	
Median (min–max)	14.2 (3.8–30.9)	52.6 (30.1–67)	157.8 (8.4–184.2)	11.7 (1–143.1)	

MLG with Poisson distribution and identity binding function.

a MLG with normal distribution and logarithmic link function.

Table 6 shows there was a statistically significant difference in the mean number of myelinated axons among all groups (p < 0.05) – it was higher in the erythropoietin group and lower in the combination group. The sum of myelinated axon lengths was higher in the erythropoietin group compared to the control group (p = 0.001) and compared to the combination group (p = 0.028) only.

**Table 6:** Multiple comparisons of immunohistochemical parameters among groups.

**Variable**	**Control**	**GM1**	**Erythropoietin**	**GM1 + Erythropoietin**	**p**
	**(n = 8)**	**(n = 8)**	**(n = 8)**	**(n = 8)**	
BMS					>0.999
Mean ± DP	9±0	9±0	9±0	9±0	
Median (min–max)	9 (9–9)	9 (9–9)	9 (9–9)	9 (9–9)	
MFS					0.451
Mean ± DP	11.4±1.2	10.5±1.1	11±1.1	11±0.9	
Median (min–max)	11 (10–13)	11 (9–12)	11 (10–13)	11 (10–13)	

ANOVA

## Discussion

Previous tests with GM1 alone and in combination with other drugs have already been performed in the study's laboratory. The authors recently demonstrated a synergistic effect between GM1 and erythropoietin in Wistar rats.^21^

It is believed that the mechanism of action of GM1 promotes a reduction in neuronal edema by increasing the activity of ion pumps, thus rebalancing cell homeostasis^23^ and, mainly, increasing endogenous neuroprotective factors, activating intracellular metabolic pathways and leading to an increase in neuronal migration, dendritic emissions, and axonal growth.^23^ On the other hand, erythropoietin promotes apoptosis blockade, modulation of the inflammatory cascade, protection, and optimization of microvascular repair, neuronal regeneration, oligodendrogenesis, and electrical activity^11^^,^^12^^,^^28^^,^^29^ further regulating intracellular calcium and the synthesis and release of neurotransmitters. However, erythropoietin has not been clearly shown to promote new synapses.^30^^,^^31^

Unlike what was found in the study with Wistar rats, in which a synergism between GM1 and erythropoietin was identified, promoting better functional neurological recovery, the authors did not find this response in BALB/c mice.^21^

In the present study, findings suggest there is a partially inhibitory action of erythropoietin on GM1. Functional assessment made by both the BMS and MFS scales showed a worse functional assessment in the group to which a combination of these two drugs was administrated when compared to the group to which GM1 was administered alone. In turn, the combination group had a better performance when compared to the group to which erythropoietin was administered alone. This difference was statistically significant as of the fourth assessment week. These findings suggest that the combination of these drugs has an inhibitory effect on neuronal regeneration when tested in mice and that erythropoietin possibly has a pharmacological action that antagonizes the effect of GM1 in promoting axonal regeneration through the site where SCI was induced. This effect varies among different mammal species, which can make it difficult to transpose an experimental study in animals to a clinical study in humans. Current literature suggests studies in mice differ from those in rats, there being differences when comparing the functional, histological, tissue repair, metabolic vascular, and regenerative responses of these animals.^32^ In rats submitted to spinal cord contusion trauma, the most important histological findings were cavitation and intramedullary cystic formation at the lesion site, whereas in mice, the most remarkable histological aspect was the presence of massive fibrosis, intense cellularity at the lesion site, and absence of cavitation. This difference in response to trauma among different species of experimental animals may partially explain the difference found in the present study in mice when compared to studies in Wistar rats. In the preliminary preparation of this work, there was an expectation that the study in mice would probably confirm data found in studies with similar designs using rats and also testing GM1 and erythropoietin in the context of spinal trauma.^20^ However, peculiarities present in regenerative responses to trauma found in mice and particularities of the mechanisms of action of GM1 and erythropoietin in tissue repair represent an investigative path to explain the differences found in this study. A plausible hypothesis considering the role of erythropoietin in blocking apoptosis, modulating the inflammatory cascade, and protecting and optimizing microvascular repair and neuronal regeneration is that, alone, it was not able to overcome the barrier formed by intense fibrosis at the injury site and, despite promoting an increase in axons and nerve fibers in this region, as identified in the immunohistochemical study, it was not able to efficiently promote nerve stimulus conduction and cell synapses able to cross this area of tissue fibrosis.

GM1 mainly promotes cell homeostasis, the presence of endogenous neuroprotective factors and axonal proliferation, dendrites, and new nerve synapses. The functional result obtained in this experiment may be related to the improved efficiency of GM1 in preserving nerve fibers in the penumbra area of the injury through the maintenance and promotion of cell homeostasis, thus preventing cell apoptosis and promoting new synapses and fiber regeneration in the contusion area. Some of the mechanisms of action of erythropoietin probably act negatively, blocking, even where partially, this positive effect of GM1 on cell preservation and regeneration. Considering that mice have intense fibrosis at the injury site when compared to rats, it is prudent to assume that erythropoietin may have its cell regeneration role attenuated in an environment of predominant tissue repair through fibrosis.

## Conclusions

This study showed that both GM1 and erythropoietin have a therapeutic action, promoting motor function recovery in BALB/c mice submitted to experimental SCI. When administered alone, GM1 led to a better functional performance of mice. The group submitted to the combined therapy had a lower functional performance compared to the GM1 group and better functional performance compared to the erythropoietin group. All groups that received GM1 and erythropoietin in combination or alone had a better functional performance than the control group, which received saline. The immunohistochemical analysis of spinal cords extracted from mice at the end of the study and sent for microscopic analysis showed a more exuberant axonal regeneration process in the erythropoietin group when compared to the other groups. Both in a number of regenerated axons and sum of fiber lengths, the erythropoietin group had a better performance.

## Author contributions

Torelli AG designed the study, collected, and analyzed the data, performed the procedures, wrote and reviewed the manuscript final version. Cristante AF performed the final review of the literature and project, analyzed the data, and reviewed the manuscript. Barros-Filho TEP performed the final review of the literature and project, designed the study, analyzed the data, and reviewed the final version of the manuscript. Santos GB analyzed the data and literature, performed the procedures, and reviewed the manuscript. Morena BC performed the immunohistochemical analysis. Correia FF performed the immunohistochemical analysis. Paschon V analyzed the data and literature, performed the immunohistochemical analysis, and reviewed the manuscript.

## Conflicts of interest

The authors declare no conflicts of interest.
